# Transcatheter Mitral Valve-in-Valve Implantation With a New Transcatheter Heart Valve for Bioprosthetic Degeneration

**DOI:** 10.3389/fcvm.2021.783507

**Published:** 2022-01-20

**Authors:** Yuntao Lu, Ye Yang, Wenshuo Wang, Jinmiao Chen, Minyan Yin, Liqi Huang, Lili Dong, Chunsheng Wang, Lai Wei

**Affiliations:** ^1^Department of Cardiac Surgery, Zhongshan Hospital, Fudan University, Shanghai, China; ^2^Shanghai Engineering Research Center of Heart Valve, Shanghai, China; ^3^Shanghai Institute of Medical Imaging, Shanghai, China; ^4^Department of Echocardiography, Zhongshan Hospital, Fudan University, Shanghai, China; ^5^Department of Cardiac Surgery, Shanghai Public Health Clinical Center, Shanghai, China

**Keywords:** bioprosthetic degeneration, valve-in-valve, J-Valve, transapical, mitral valve

## Abstract

**Background:**

Transcatheter mitral valve-in-valve (TMVIV) procedure with aortic transcatheter heart valves has recently become a less invasive alternative for patients with mitral bioprosthetic dysfunction. This study reports the initial experience of TMVIV implantation using the J-Valve System (JieCheng Medical Technology Corporation Ltd., Suzhou, China).

**Methods:**

A retrospective observational multicenter study was conducted to evaluate the short-term outcomes of TMVIV. In total, 26 consecutive patients with symptomatic bioprosthetic failure at eight hospitals underwent TMVIV using the J-Valve System between May 2019 and June 2021. Procedural results and clinical outcomes were analyzed using the Mitral Valve Academic Research Consortium criteria.

**Results:**

The mean age was 75.3 ± 7.1 years and 69.2% of patients were female. The mean Society of Thoracic Surgeons Predicted Risk of Mortality score was 12.3 ± 8.3%. The technical success rate was 96.2%. Nine of the 26 patients (34.6%) were implanted with a J-Valve of a size equal to the internal diameters of the deteriorated prostheses. At the 30-day and 1-year follow-ups, all-cause mortality was 3.8 and 16.0% and the stroke rates were 0 and 12.0%, respectively. Device-related mortality was 0% and the mean mitral valve gradient was 6.4 ± 2.7 mm Hg. No patient experienced device embolization, left ventricular outflow tract obstruction, or mitral valve reintervention. Postprocedural mitral regurgitation was none or trace in all the patients. All the patients were in the New York Heart Association (NYHA) class ≤ II at the last follow-up.

**Conclusion:**

Transcatheter implantation of the J-Valve System in high-risk patients with mitral bioprosthetic dysfunction was found to be a reasonable alternative and associated with good short-term outcomes.

## Introduction

Mitral valve disease is the most prevalent form of valvular disease, affecting 10% of patients over the age of 75 years (1). Bioprosthetic valves have become more common in the treatment of mitral valve disease. Consequently, structural valve deterioration is the most prevalent problem and reoperation is required in as many as 35% of patients within the first 10 years after mitral valve surgery (2). Redo mitral valve surgery is associated with high perioperative morbidity and mortality (3, 4) due to repeat sternotomy, cardiopulmonary bypass, the older age of patients, and severe comorbidities. Nevertheless, transcatheter mitral valve-in-valve (TMVIV) implantation has been developed as a feasible and safe treatment for high-risk and inoperable patients (5–7). In TMVIV, an oversizing strategy is preferred due to the risk of embolization resulting from high gradient pressure between the ventricle and atrium. However, excessive oversizing may be unfavorable, as it leads to under expansion of the transcatheter heart valve (THV) device, which increases the risk of leaflet pin-wheeling, device thrombosis, and decreased durability (8).

**Figure 1:**
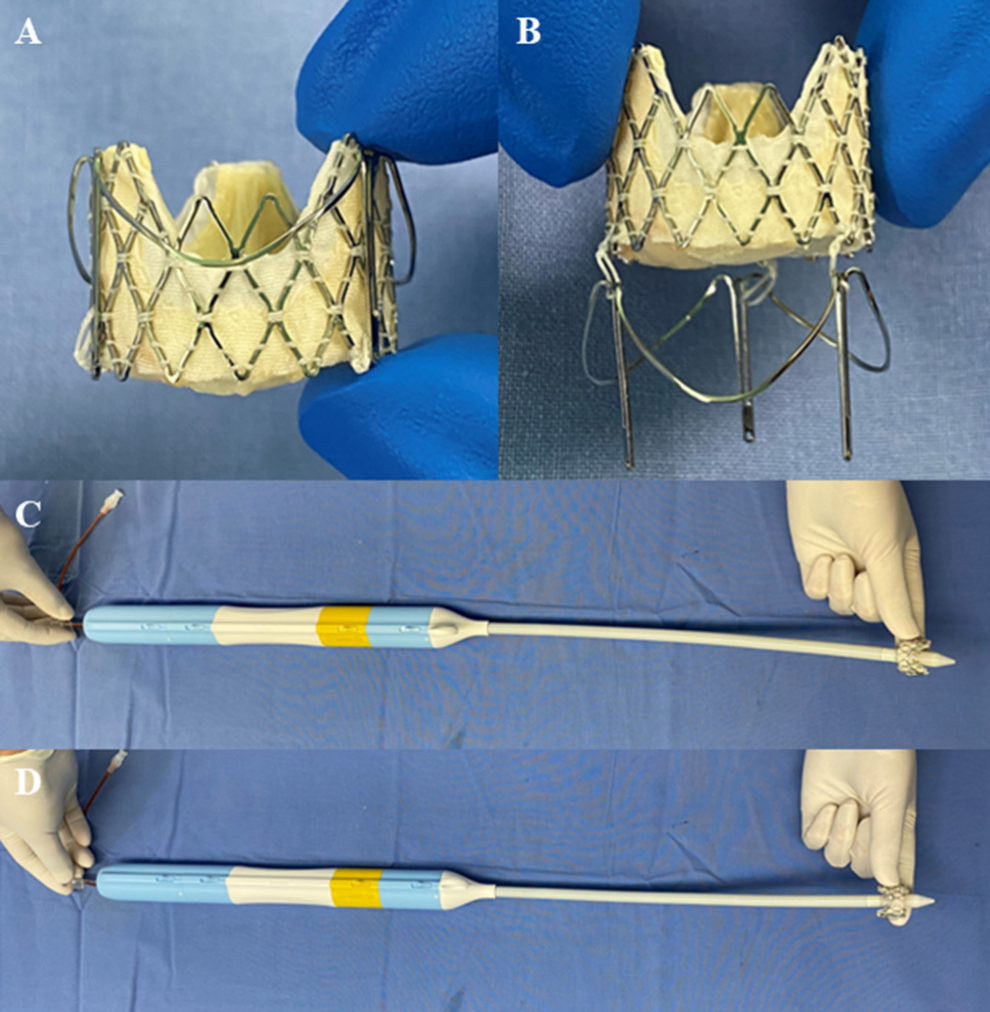
The J-Valve system (JieCheng Medical Technology Corporation Ltd., Suzhou, China). **(A)** The prosthesis was combined with locators after release. **(B)** Movable connection between prosthesis and locators. **(C)** Prosthesis orientation for the transapical aortic valve replacement using the J-Valve system. **(D)** Prosthesis orientation for the transapical mitral valve-in-valve implantation using the J-Valve system.

The J-Valve System (JieCheng Medical Technology Corporation Ltd., Suzhou, China) is a low-profile, self-expanding THV (Figures 1A,B). Excellent short-term outcomes, such as 4.7% all-cause mortality, 2% new permanent pacemaker implantation, 0% coronary artery obstruction, and 0% myocardial infarction at the 1-year follow-up, have demonstrated the efficacy and safety of the J-Valve in the treatment of patients with aortic stenosis and/or insufficiency (9–12). While the J-Valve was originally designed to treat aortic stenosis and/or insufficiency (12), its specific self-positioning design is also favorable in TMVIV implantation. Inspired by the Sapien prosthesis (Edwards Lifesciences Incorporation, Irvine, California, USA) to initially perform transapical mitral valve-in-valve by crimping the valve into the delivery catheter in the opposite direction, we attempted to treat high-risk or inoperable patients with degenerative mitral bioprostheses using the J-Valve System.

## Methods

### Patient Population

We conducted a retrospective observational analysis for all the consecutive patients who underwent TMVIV with the J-Valve System for the treatment of a degenerated mitral bioprosthesis at eight medical centers between May 2019 and June 2021. Indications for redo mitral valve replacement were based on the 2014 American College of Cardiology/American Heart Association Guideline for the Management of Patients with Valvular Heart Disease (13). All the patients were evaluated by a multidisciplinary heart team and found to have high surgical risk scores and/or severe comorbidity precluding redo valve surgery with cardiopulmonary bypass. The only exception was a 50-year-old patient whose symptoms could not be controlled by drugs. We strongly advised him to choose conventional surgery, but he still declined it and opted for TMVIV surgery as the preferred choice. The exclusion criteria for the TMVIV procedure were active endocarditis, prosthetic valve endocarditis, left atrial and/or left ventricular thrombosis, moderate or severe mitral paravalvular leakage, a true internal diameter (ID) of mitral bioprostheses <20 mm, a requirement for concomitant coronary artery bypass graft, and high risk for TMVIV-induced left ventricular outflow tract (LVOT) obstruction.

### Ethics

All the patients or their legal representatives were fully informed about the procedure and signed written consent prior to surgery. The study protocol was approved by the Ethics Committee of Zhongshan Hospital, Fudan University.

### Devices

The J-Valve System is composed of a bioprosthetic valve and a transapical delivery catheter (Figure 1C). The bioprosthetic valve is a porcine valve supported by a self-expanding nitinol structure of different sizes: external diameters of 21, 23, 25, 27, and 29 mm. The size of the J-Valve mentioned below refers to the external diameter. A set of 3 “U” -shaped nitinol hoops were designed to surround the aortic valve as locators to position the device to sit in three aortic sinuses to facilitate accurate positioning of the implanted valve and fix it to the native valve (10). Valve sizes of 21, 23, and 25 mm were crimped into the 27 F delivery catheter and 33 F catheter for valve sizes of 27 and 29 mm.

### Procedures

Before the procedure, all the patients underwent transthoracic echocardiography and contrast-enhanced multislice CT to assess (1) the severity and types of bioprosthetic failure; (2) the bioprosthesis dimensions for the sizing of the J-Valve; (3) the mitral valve, left ventricle, and aortic root anatomy to evaluate the risk of LVOT obstruction; and (4) the coronary vessels or bypass grafts for significant coronary artery disease.

**Figure 2:**
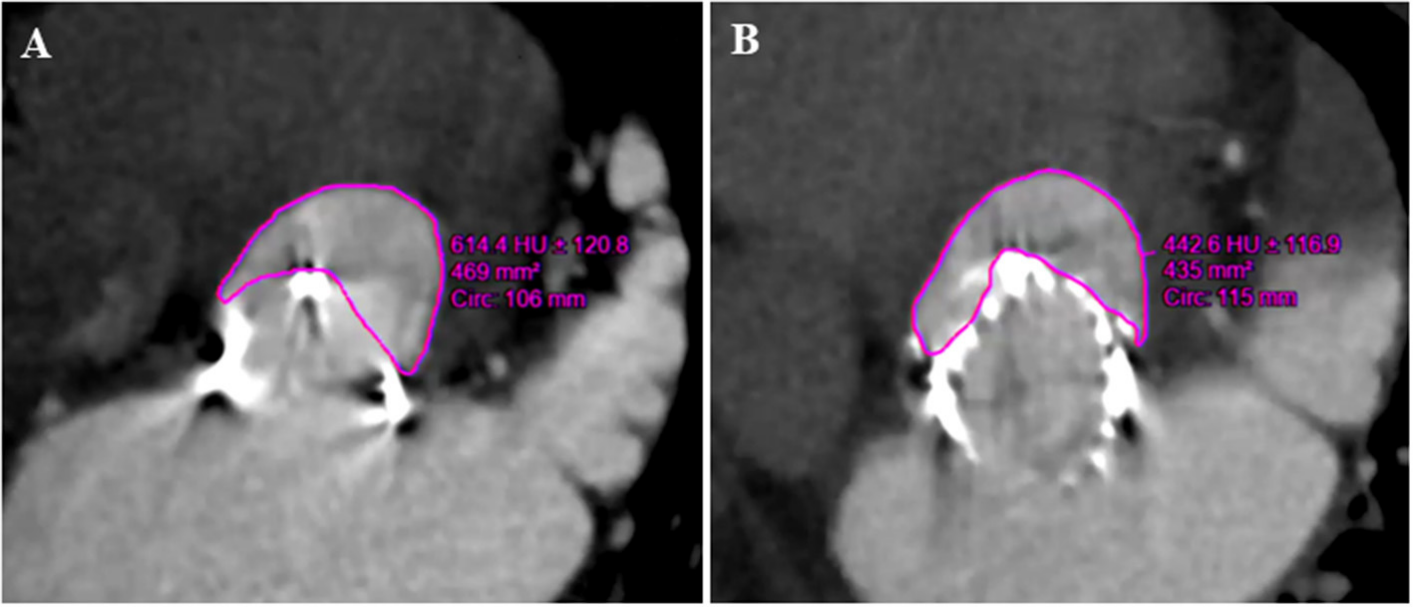
Preoperative and postoperative multidetector CT in the assessment of neo-LVOT (Patient number 23). **(A)** The predicted area of neo-LVOT was 469 mm^2^ before the J-Valve implantation. **(B)** The postoperative area of neo-LVOT was 435 mm^2^. LVOT, left ventricular outflow tract.

The neo-LVOT surface area was estimated on CT images in systole (Figure 2A) using Vitrea software (version 6.5.3, Vital Images Incorporation, Minnetonka, Minnesota, USA). We used a predicted surface area <200 mm^2^ as a cutoff value to identify patients at risk for TMVIV-induced LVOT obstruction.

The size of the J-Valve and the balloon used for valvuloplasty were selected based on the true ID of the surgical heart valves (SHVs) from CT measurements and/or the “valve-in-valve” app according to the manual of the manufacturer. In 15 patients, we oversized the implanted valves. A 23-mm J-Valve was implanted in only one patient with an SHV ID measuring 25 mm because of severe calcification of the leaflets. For the remaining patients, we implanted the J-Valve with the strategy of “true sizing” meaning that the size of the THV is equal to the ID of the SHV. The J-Valve prosthesis and three locators were preloaded into the delivery system direction opposite to that used for aortic valve replacement (Figures 1C,D).

**Figure 3:**
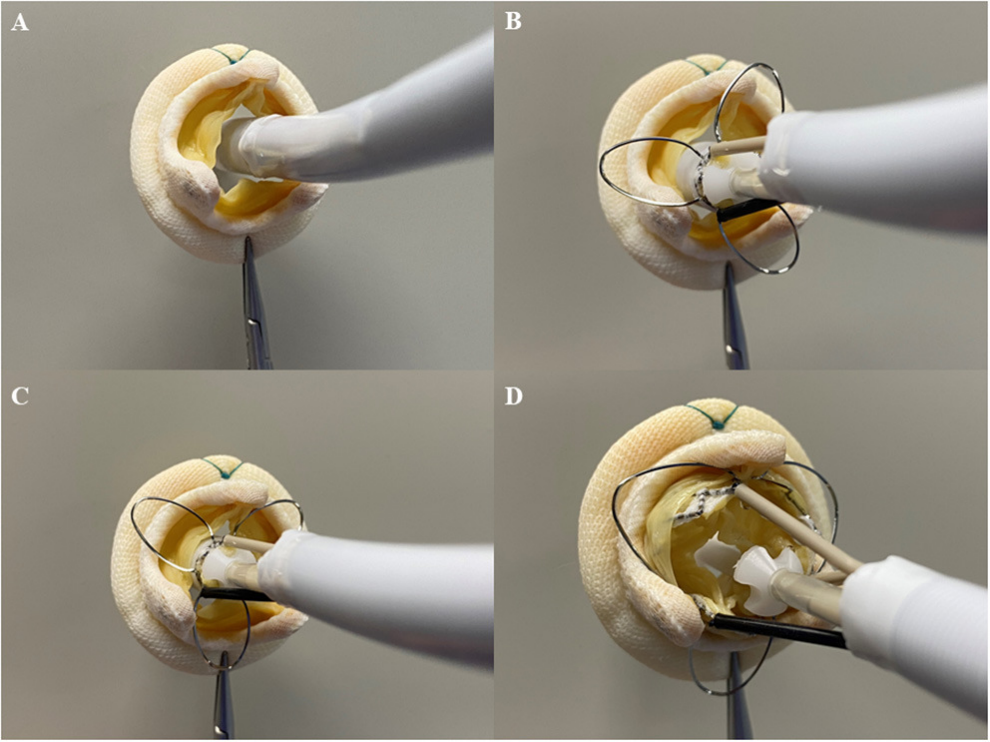
Process of transapical mitral valve-in-valve implantation with the J-Valve system *in vitro*. **(A)** The delivery system was inserted into the surgical mitral prosthesis. **(B)** The locators were released. **(C)** The locators were placed in “sinuses” of the surgical mitral prosthesis. **(D)** The transcatheter prosthesis was released and the surgical mitral prosthesis was fixed between the transcatheter prosthesis and locators.

With patients under general anesthesia, all the surgeries were performed in a hybrid operating room with cardiopulmonary bypass on standby. During the procedure, the C-arm was directed at a specific angle, so that any two of the three stent posts of the SHV totally overlapped under fluoroscopy. The transapical approach was used in all the cases. A limited left thoracotomy was made. Two 3–0 polypropylene (Ethicon, Somerville, New Jersey, USA) Teflon-reinforced mattress sutures were placed on the left ventricular apex and the patient was administered heparin to maintain an activated clotting time > 250 s. A guidewire was inserted from the middle of the suture to reach the left atrium through the SHV. Balloon valvuloplasty was performed only in cases of mitral bioprosthetic stenosis before J-Valve implantation during rapid ventricular pacing (160–180 beats/min). The J-Valve delivery system was then inserted into the left ventricle and atrium *via* a guidewire. The three locators were released first and the delivery catheter was gently advanced toward the atrium to help the three locators accurately sit in the SHV “sinuses” among the three struts. Then, the J-Valve was released and deployed with the aid of the locators, so that it was fixed in the middle of the SHV after self-expansion (Figure 3). Finally, postimplant balloon valvuloplasty was performed in all the cases to ensure that the THV fully fit within the SHV (Supplementary Video). A vitamin K antagonist was initiated on the day following the procedure with a target international normalized ratio of 2 to 3 for 6 months.

### Definitions and Study Endpoints

We used standardized endpoint criteria according to the Mitral Valve Academic Research Consortium (MVARC) for the data collection (14). The endpoints of this study included technical success at the exit from the procedure room as well as all-cause mortality. Other clinical endpoints, including device-related mortality, device embolization (the device moves during or after deployment such it loses contact with its initial position), LVOT obstruction, echocardiographic hemodynamic parameters, access site complications, myocardial infarction, stroke, permanent pacemaker implantation, bleeding, acute kidney injury, and rehospitalization at the 30-day follow-up and last clinical follow-up, were also evaluated. LVOT obstruction in this study was defined as a severe hemodynamic compromise. In addition, we collected data on procedure details, length of postprocedural hospital stay, and the New York Heart Association (NYHA) class at 30 days and last follow-up.

All the patients underwent transesophageal echocardiography examinations during the procedure and were followed-up using transthoracic echocardiography at discharge, 1 month, 3–6 months, 1 year, and once every year.

### Statistical Methods

Categorical variables are expressed as numbers and percentages. Normal variables are expressed as the mean ±SD. Nonnormally distributed parameters are presented using medians (interquartile ranges). The Kaplan–Meier survival curves were used to analyze survival. Statistical analysis was conducted using the SPSS software (version 20.0; SPSS Incorporation, Chicago, Illinois, USA).

## Results

### Baseline Clinical Characteristics

Between May 2019 and June 2021, 26 consecutive patients (18 female; mean age 75.3 ± 7.1 years) with symptomatic bioprosthetic mitral valve dysfunction (regurgitation and/or stenosis) at eight centers underwent TMVIV. The indications for the TMVIV procedure were severe prosthetic stenosis in four patients, severe regurgitation in 21 patients, or a combination of stenosis and regurgitation in one patient. The mean Society of Thoracic Surgeons Predicted Risk of Mortality score was 12.3 ± 8.3%. Furthermore, the mean pulmonary artery systolic pressure was 61.8 ± 17.3 mm Hg and the mean left ventricular ejection fraction was 63.4 ± 5.8%. All the patients had heart failure symptoms with the NYHA classification III or IV at admission. The baseline characteristics of 26 patients are shown in Table 1.

**Table 1:** Baseline clinical characteristics.

**Variables**	***N* = 26**
Age, years	**75.3** **±** **7.1**
Female	**18 (69.2)**
Body mass index, Kg/m^2^	**22.8** **±** **3.6**
NYHA class III	**15 (57.7)**
NYHA class IV	**11 (42.3)**
STS, %	**12.3** **±** **8.3**
Hypertension	**16 (61.5)**
Diabetes mellitus	**6 (23.1)**
Stroke	**3 (11.5)**
Atrial fibrillation	**17 (65.4)**
Chronic lung disease	**1 (3.8)**
Anemia	**8 (30.8)**
Prior CABG	**1 (3.8)**
Second redo cardiac surgery	**1 (3.8)**
Pulmonary Edema	**2 (7.7)**
ECMO	**1 (3.8)**
Emergency surgery	**3 (11.5)**
Mitral bioprosthetic dysfunction	
Duration, years	**11.0** **±** **2.6**
Regurgitation	**21 (80.8)**
Stenosis	**4 (15.4)**
Combination	**1 (3.8)**
Tricuspid regurgitation	
Moderate	**7 (26.9)**
Severe	**9 (34.6)**
LA diameter, mm	**54.8** **±** **10.0**
PASP, mmHg	**61.8** **±** **17.3**
LVEF, %	**63.4** **±** **5.8**

*NYHA, New York Heart Association; STS, Society of Thoracic Surgeons; CABG, coronary artery bypass grafting; ECMO, extracorporeal membrane oxygenation; LA, left atrium; PASP, pulmonary artery systolic pressure; LVEF, left ventricular ejection fraction. Values are mean ± SD or n (%)*.

### Procedure Details

Detailed characteristics of the failed bioprostheses and valve-in-valve procedure are shown in Table 2. The average duration from surgical valve replacement to bioprosthetic failure was 11.0 ± 2.6 years. Transapical valve-in-valve implantation was performed for all the patients. Balloon dilatation was performed in five prosthetic stenosis cases before J-Valve implantation (patient numbers 3, 4, 10, 13, and 15), and balloon dilatation was performed in all the patients after J-Valve implantation.

**Table 2:** Detailed characteristics of the failed bioprostheses and valve-in-valve procedure.

**Pt**	**Failed prosthesis type**	**Age year**	**Failure Mode**	**Label Size mm**	**True ID mm**	**S3 size by app**	**J-Valve size mm**	**Oversizing** **mm**		**Balloon** **Dilatation**		**Peak/Mean** **transvalvular** **gradient, mmHg**		**MR grade** **(0–4)**	
								**S3**	**J-Valve**	**Pre**	**Post**	**Pre**	**Post**	**Pre**	**Post**
1	Hancock II	10	MR	27	22	23	25	+1	+3	NO	YES	34/14	10**/**5	4	1
2	Perimount	10	MR	25	23	26	23	+3	0	NO	YES	29/14	14/5	4	1
3	Hancock II	10	MS	27	22	23	25	+1	+3	YES	YES	50/27	18/9	2	1
4	Epic	10	MS	29	25	26	27	+1	+2	YES	YES	45/21	11/7	1	0
5	Hancock II	12	MR	27	22	23	23	+1	+1	NO	YES	25/9	10/6	4	1
6	CE Standard	7	MR	27	23	26	23	+3	0	NO	YES	27/14	10/6	4	1
7	Hancock II	10	MR	29	24	26	25	+2	+1	NO	YES	NA	7/5	4	1
8	Hancock II	13	MR	29	24	26	25	+2	+1	NO	YES	21/9	7/3	4	1
9	Hancock II	11	MR	27	22	23	25	+1	+3	NO	YES	38/NA	28/NA	4	0
10	Perimount	15	MS	27	25	26	25	+1	0	YES	YES	41/NA	14/NA	2	1
11	Hancock II	11	MR	27	22	23	25	+1	+3	NO	YES	NA	6/4	4	0
12	Hancock II	10	MR	29	24	26	25	+2	+1	NO	YES	29/6	11/4	4	0
13	Perimount	17	MS + MR	27	25	26	23	+1	−2	YES	YES	29/NA	9.0/NA	2	1
14	Epic	14	MR	29	25	26	25 + 23	+1	0	NO	YES	38/13	22/9	4	1
15	Perimount	15	MS	27	25	26	25	+1	0	YES	YES	40/23	6/3	1	1
16	CE Standard	9	MR	29	25	26	25	+1	0	NO	YES	15/7	6/4	4	1
17	Epic	8	MR	27	23	26	25	+3	+2	NO	YES	21/9	7/4	4	1
18	Epic	11	MR	27	23	26	23	+3	0	NO	YES	19/8	12/7	4	1
19	Hancock II	11	MR	27	22	23	23	+1	+1	NO	YES	19/7	10/5	4	1
20	Hancock II	10	MR	29	24	26	25	+2	+1	NO	YES	NA	NA/4	4	1
21	Hancock II	13	MR	29	24	26	25	+2	+1	NO	YES	13/7	9/4	4	1
22	Epic	6	MR	27	23	26	23	+3	0	NO	YES	16/9	11/6	4	1
23	Perimount	15	MR	27	25	26	25	+1	0	NO	YES	16/7	13/5	4	1
24	Hancock II	10	MR	27	22	23	23	+1	+1	NO	YES	18/10	16/9	4	1
25	CE Standard	8	MR	29	25	26	25	+1	0	NO	YES	29/9	10/4	4	1
26	Hancock II	10	MR	25	20.5	23	23	+2.5	+2.5	NO	YES	16/7	13/9	4	1

*PT, patient; ID, internal diameter; S3, Sapien 3 (Edwards Lifesciences Incorporation, Irvine, California, USA); THV, transcatheter heart valve; MR, mitral regurgitation; CE, Carpentier-Edwards; NA, not available; MS, mitral stenosis; grade (0–4): 0 = none; 1 = trace; 2 = mild; 3 = moderate; 4 = severe*.

Other procedural results and 30-day outcomes are shown in Table 3. The technical success rate was 96.2%, as defined by the MVARC. One patient (patient number 14) needed second J-Valve implantation because one prolapsed leaflet of the SHV occluded the inflow tract after the first J-Valve implantation. Transcatheter aortic valve replacement (TAVR) with the J-Valve was simultaneously performed in one patient (patient number 14).

**Table 3:** Procedural details and 30-day outcomes.

**Variables**	***N* = 26**
Procedural details	
Transapical access	26 (100)
J-Valve	26 (100)
THV size strategy
Oversizing	15 (57.7)
“True sizing”	9 (34.6)
Downsizing	1 (3.8)
MVARC technical success	25 (96.2)
Simultaneously TAVR	1 (3.8)
Contrast dose, ml	40 (20,140)
Procedural complications	1 (3.8)
Conversion to surgery	0
Need for second THV implantation	1 (3.8)
Dislocation	0
LVOT obstruction	0
Left ventricular perforation	0
30-day outcomes	
Peak MVG, mmHg	11.1 ± 5.1
Mean MVG, mmHg	5.8 ± 2.7
Mitral valve regurgitation ≥ mild	0
Death	1 (3.8)
Device-related death	0
Myocardial infarction	0
Stroke	0
Permanent pacemaker implantation	0
Access site complication	1 (3.8)
Life-threatening Bleeding	1 (3.8)
Acute kidney injury	1 (3.8)
Stage 1	1 (3.8)
Stage 2 or 3	0
Length of post-procedural hospital stay, days	8 (4,30)
Cardiovascular rehospitalization	0
Noncardiovascular rehospitalization	1 (3.8)
NYHA class ≥ III	0

*THV, transcatheter heart valve; “true size: the size of transcatheter heart valve equal to the internal diameter of a deteriorated prosthesis; MVARC, Mitral Valve Academic Research Consortium; TAVR, transcatheter aortic valve replacement; LVOT, left ventricular outflow tract; MVG, mitral valve gradient; NYHA, New York Heart Association. Values are mean ± SD, n (%) or median (min, max)*.

### 30-Day Outcomes

One patient (3.8%) died of pulmonary infection 8 days after the TMVIV procedure. No device embolization, LVOT obstruction, mitral valve reintervention, or neurological complications occurred. One patient (3.8%) developed acute kidney injury and renal function had recovered at discharge. One patient (3.8%) needed surgery *via* the left thoracic incision for life-threatening bleeding. One patient (3.8%) was rehospitalized because of pneumonia. The median length of the postprocedural hospital stay was 8 days (range, 4–30). Heart failure symptoms were significantly reduced and all the patients were at the NYHA II or less at the 30-day follow-up.

The peak mitral valve gradient (MVG) was 11.1 ± 5.1 mm Hg and the mean MVG was 5.8 ± 2.7 mm Hg. In total, 14 of 23 (60.9%) patients had a mean gradient of 5 mm Hg or less. The severity of paravalvular leakage or regurgitation was ≤ mild for all the patients. Notably, all the patients showed heart function improvement and were in the NYHA class ≤ II.

### 1-Year Follow-Up Outcomes

**Figure 4:**
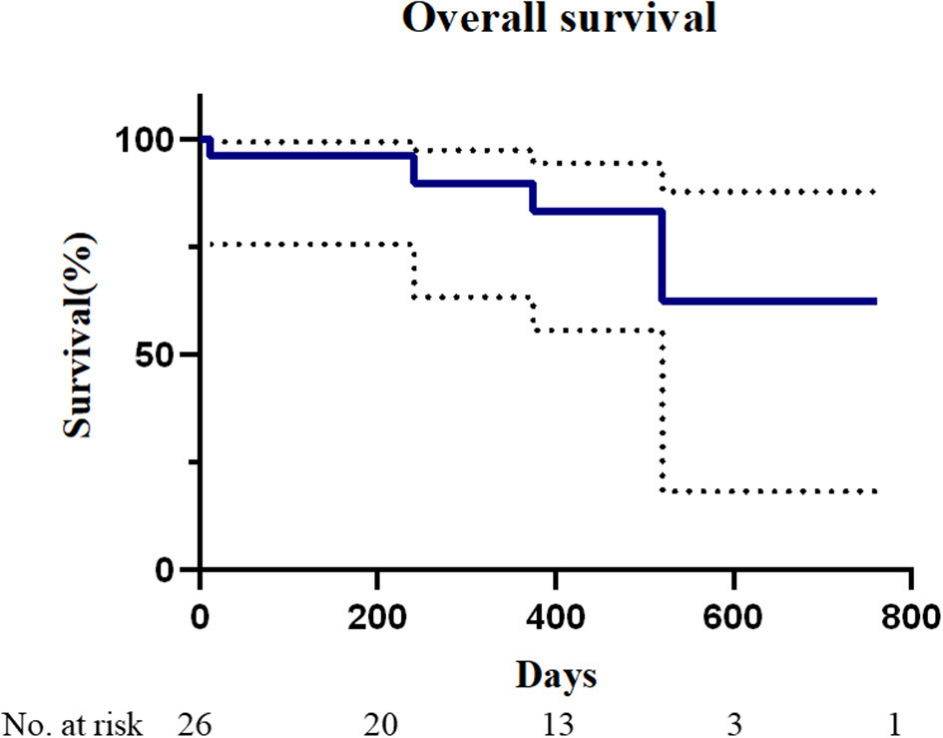
Mortality after transapical mitral valve-in-valve implantation. The Kaplan–Meier analysis of overall survival in patients who underwent transapical mitral valve-in-valve (*n* = 26).

The median follow-up time was 370 days (range, 8–762 days). Only one patient had a postoperative follow-up time of less than 3 months. All-cause mortality was 16.0% and device-related mortality was 0%. The overall survival during follow-up is given in Figure 4. One patient (patient number 11) developed a hemorrhagic stroke from vitamin K antagonist overdose 251 days after TMVIV and she died of central nervous system infection 15 days after emergency lateral ventricular drainage. Another patient (patient number 13) with atrial fibrillation died of severe hemorrhagic stroke 375 days after TMVIV. One patient (patient number 3) died of pneumonia 520 days after implantation. One patient (patient number 5) with atrial fibrillation developed ischemic stroke from insufficient anticoagulation 228 days after TMVIV and had recovered without disability at the last visit. One patient (patient number 2) needed rehospitalization for tachycardia from atrial fibrillation. No patient experienced valve-related reintervention, device embolization, myocardial infarction, dialysis, or new pacemaker implantation.

The peak MVG was 16.9 ± 5.2 mm Hg and the mean MVG was 6.4 ± 2.7 mm Hg. Postprocedural mitral regurgitation was none or trace in all the patients. The mean left ventricular ejection fraction was 63.2 ± 4.6% and all the patients were in the NYHA class ≤ II. The last follow-up clinical outcomes are shown in Table 4.

**Table 4:** One-year outcomes.

**Variables**	***N* = 25**
All-cause death	4 (16.0)
Device-related death	0
Stroke	3 (12.0)
Ischemic stroke	1 (4.0)
Hemorrhagic stroke	2 (8.0)
Access site complication	1 (4.0)
Valve-related reintervention	0
Device embolization	0
New dialysis requirement	0
New pacemaker implantation	0
Mitral valve regurgitation ≥ mild	0
Peak MVG, mmHg	16.9 ± 5.2
Mean MVG, mmHg	6.4 ± 2.7
LVEF,	63.2 ± 4.6
PASP, mmHg	42.4 ± 10.4
Cardiovascular rehospitalization	1 (4.0)
NYHA class ≥ III	0

*MVG, mitral valve gradient; LVEF, left ventricular ejection fraction; PASP, pulmonary artery systolic pressure; NYHA, New York Heart Association. Values are mean ± SD or n (%)*.

## Discussion

This study demonstrates the feasibility of the J-Valve for use in TMVIV in patients with degenerated mitral bioprostheses to treat mitral regurgitation, mitral stenosis, or a combination of the two. The MVARC-defined technical success rate with the J-Valve was 96.2%, which is comparable to that reported in the TMVIV multicenter registry study of 94.6% (7). TMVIV using the J-Valve was also associated with good short-term outcomes. This study showed 3.8% all-cause mortality at the 30-day follow-up and 16.0% at 1-year follow-up, whereas the 30-day and 1-year all-cause mortality in the Transcatheter Valve Therapy Registry (5) were 5.4 and 16.7%, respectively. The stroke rates were 0 and 12.0% at the 30-day and 1-year follow-ups, respectively, and 1.1 and 3.3% in the previous report (5). No device-related death, device embolization, or LVOT obstruction occurred. All the patients experienced a clinically important improvement in heart failure symptoms during follow-up. The hemodynamic performance was acceptable after the TMVIV procedure with the J-Valve. The mean MVG was 5.8 mm Hg and 6.4 mm Hg at the 30-day and 1-year follow-ups, respectively, which is comparable to those in the TMVIV multicenter registry study of 7.3 mm Hg and 7.0 mm Hg, respectively (7). No patient had more than mild mitral regurgitation or required valve-related reintervention after TMVIV during follow-up.

In the TAVR procedure, the J-Valve System can fix the native aortic valves in the middle of its locators and frame, which can offer robust support to reduce the risk of left ventricular dislocation (10, 11). In addition, the J-Valve is the low-profile THV that is suitable for TMVIV implantation. In *in vitro* test, the locators could be deployed in the “sinuses” of the SHVs (Figure 3). The construction of the J-Valve with the locators could also reduce the risk of embolization, as it works in the aortic position. Inspired by these features, we initially applied the J-Valve in the TMVIV procedure as a second choice in the treatment of high-risk surgical patients with mitral bioprosthetic deterioration.

**Figure 5:**
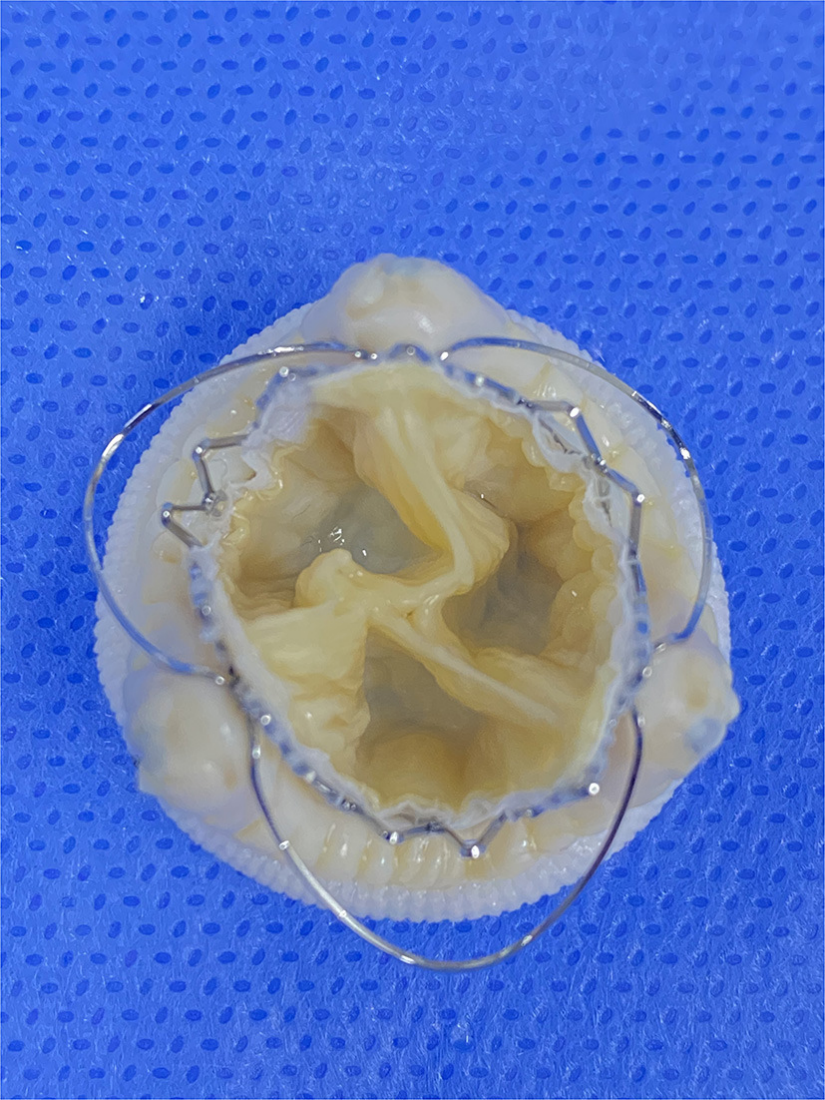
The oversizing transcatheter heart valves implantation inside different surgical heart valves *in vitro*. Pinwheel-like leaflets of underexpanded transcatheter heart valves. A 23-mm J-Valve inside a 25-mm epic (internal diameter of 21 mm).

**Figure 6:**
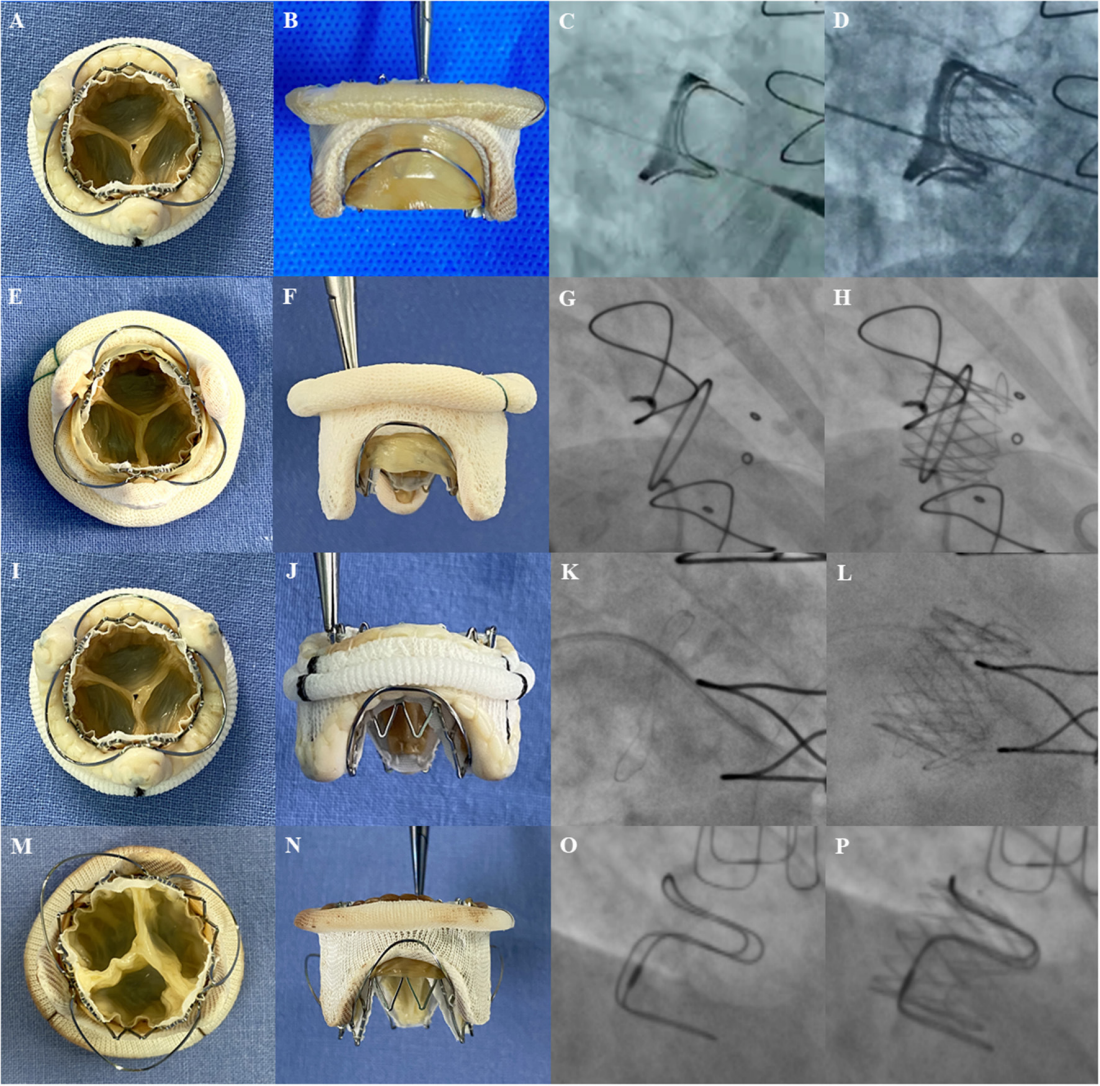
The transcatheter heart valves implantation inside the different surgical heart valves. Photos of surgical bioprosthetic mitral valves with J-Valve implantation *in vitro* (left 2 panels) and fluoroscopic images before (third panel) and after (fourth panel) implantation. **(A,B)** A 23-mm J-Valve inside a 25-mm Perimount (internal diameter of 23 mm). **(C,D)** A 25-mm J-Valve inside a 27-mm Perimount (internal diameter of 25 mm); **(E,F)** A 21-mm J-Valve inside a 25-mm Mosaic (internal diameter of 20.5 mm); **(G,H)** A 25-mm J-Valve inside a 29-mm Hancock II (internal diameter of 24 mm); **(I,J)** A 21-mm J-Valve inside a 25 mm Epic (internal diameter of 21 mm); **(K,L)** A 23-mm J-Valve inside a 27-mm Epic (internal diameter of 23 mm). **(M,N)** A 25-mm J-Valve inside a 29-mm Carpentier-Edwards porcine (internal diameter of 25 mm). **(O,P)** A 25-mm J-Valve inside a 29-mm Carpentier-Edwards supra-annular valve (internal diameter of 25 mm).

Currently, the Sapien 3 (Edwards Lifesciences Incorporation, Irvine, California, USA) is used as the standard THV for most TMVIV interventions with good short-term outcomes (7, 15). To avoid embolization, it is important to avoid parallel deployment and achieve conical deployment with an oversizing strategy (8). Unlike with the Sapien XT (Edwards Lifesciences Incorporation, Irvine, California, USA) (16), interventionists tend to select the proper Sapien 3 size selection following a slightly oversizing principle for its favorable extensibility. In the early exploration of the J-Valve system, interventionalists also performed the TMVIV procedure using an oversizing strategy that would lead to under expansion of the leaflets (Figure 5). Nevertheless, the J-Valve can be sized according to the ID of the SHV for the robust fixation of locators to reduce the risk of dislocation (Figures 6A–P). In this analysis cohort, 34.6% of patients underwent TMVIV according to the principle of “true sizing.” Notably, 13 of the deteriorated SHVs in these patients were the Hancock II (Medtronic, Minneapolis, Minnesota, USA) whose label sizes of 29, 27, and 25 mm were corresponded to IDs of 24, 22, and 20.5 mm, respectively. Therefore, “true sizing” was unlikely to occur in patients with a Hancock II (Table 2). When the SHVs of the Hancock II were excluded, nine of 13 patients (69.2%) underwent TMVIV with the principle of “true sizing” (Table 2). No patient had experienced valve dislocation by the last follow-up. Therefore, TMVIV with a J-Valve system of “true sizing” may be feasible and would theoretically result in fully unfolded leaflets (Figures 6A,I,M), a lower gradient, and longer durability.

Left ventricular outflow tract obstruction is a potentially disastrous complication of TMVIV and predicting this condition still poses a challenge. The expected neo-LVOT area measured from cardiac CT is used to assess the risk of LVOT obstruction caused by transcatheter mitral valve replacement (17, 18). All the patients in this study underwent multislice CT to evaluate the risk of LVOT obstruction and no patient in this study had a predicted neo-LVOT area <200 mm^2^ (Figures 2A,B).

At the time of surgical implantation, most surgeons prefer orienting SHVs such that two posts straddle the LVOT rather than the posterior annulus so that there is no strut occluding the LVOT. However, the SHV orientation does not influence the risk of LVOT obstruction during a TMVIV procedure with Sapien valves because the height of the skirt is lower than the leaflets of the SHV (19). In contrast, the frame and skirt of the J-Valve are wavelike (Figure 1A) and with the fixation of the locators, the struts of the J-Valve usually overlap the struts of the SHV (Figures 6B,F,J,N). This characteristic would not increase the risk of LVOT obstruction. However, the length of the bovine pericardial leaflets of the Perimount (SHV; Edwards Lifesciences Incorporation, Irvine, California, USA) is longer than J-Valve's frame (Figure 6B) or the other THVs, which might increase the risk of LVOT obstruction.

Previous studies have demonstrated transfemoral MVIV to be safer than transapical implantation with fewer complications and lower mortality (7, 15). The J-Valve system was primarily designed for transapical TAVR and the transfemoral J-Valve system is still under clinical trials. We had to perform the procedure *via* transapical access rather than transvenous access. Similar to transapical aortic valve replacement, transapical TMVIV offers short and straight access to the degenerated SHV with good coaxial alignment. However, the transapical approach in this study still promoted less safe 30-day outcomes than the transfemoral approach in a large TMVIV study (7).

A previous study by Sung-Han Yoon et al. showed a high incidence of valve thrombosis after TMVIV (15). Nevertheless, anticoagulation therapy after TMVIV surgery is still controversial. Given the high proportion of atrial fibrillation (65.4%) in this cohort and the complex configuration of valve-in-valve, anticoagulation with warfarin was recommended for these patients for at least 6 months. However, two patients had a hemorrhagic stroke and one patient had an ischemic stroke during a 1-year follow-up. A retrospective review of the visit data of the three patients revealed that the two patients with hemorrhagic stroke both failed to regularly monitor prothrombin time. This may be the main reason why the stroke rate is obviously higher than that reported in other studies (7, 15) and it also suggests that anticoagulation education for the elderly should be improved.

Although TMVIV is becoming a promising therapy in high-risk patients with mitral bioprosthetic dysfunction and should be considered a therapeutic option (7), patients with small SHVs or low-risk patients may likely undergo redo surgical mitral valve replacement. This is because elevated valve gradients, the durability of the THVs, the optimal management of concomitant TR, and optimal anticoagulation strategies are still unknown and require further study (20).

## Limitation

In addition to the inherent bias of observational studies, the major limitation was that this study had a small sample size and short-term follow-up, as it included only 26 patients with a median follow-up time of 370 days (range, 8–762 days). Moreover, the leaflets of the surgical valves in Figure 4 did not deteriorate. Thus, these simulations could not authentically represent valve-in-valve implantation *in vivo*.

## Conclusion

Transcatheter implantation of the J-Valve system within a degenerated mitral bioprosthesis *via* the transapical route is feasible and associated with good short-term outcomes in high-risk surgical patients. All the patients experienced improvements in heart failure after discharge. Owing to the locator units, the J-Valve system may be a good alternative to the TMVIV procedure and its use may shed light on new devices customized for valve-in-valve procedures.

## Data Availability Statement

The original contributions presented in the study are included in the article/Supplementary Material, further inquiries can be directed to the corresponding authors.

## Ethics Statement

The studies involving human participants were reviewed and approved by Ethics Committee of Zhongshan Hospital, Fudan University. The patients/participants provided their written informed consent to participate in this study.

## Author Contributions

YL, YY, WW, JC, CW, and LW contributed to the study conception and design. MY, LH, and LD performed material preparation, data collection, and analysis. YL, YY, and JC wrote the draft of the manuscript. All the authors have read and approved the final version of the manuscript.

## Funding

This study was supported by the National Natural Science Foundation of China (Grant Number: 81974272) and Shanghai Engineering Technology Research Center for Cardiac Intervention (Grant Number: 19DZ2250300).

## Conflict of Interest

The authors declare that the research was conducted in the absence of any commercial or financial relationships that could be construed as a potential conflict of interest.

## Publisher's Note

All claims expressed in this article are solely those of the authors and do not necessarily represent those of their affiliated organizations, or those of the publisher, the editors and the reviewers. Any product that may be evaluated in this article, or claim that may be made by its manufacturer, is not guaranteed or endorsed by the publisher.

## Supplementary Material

Supplementary Video Transapical mitral valve-in-valve using the J-Valve system. The J-Valve delivery system was inserted into the left ventricle and atrium *via* a guidewire. The three locators were released first and the delivery catheter was gently advanced toward the atrium to help the three locators accurately sit in the “sinuses” of the surgical heart valve among the three structs. Then, the J-Valve was released and deployed with the aid of the locators, so that the J-Valve was fixed in the middle of the surgical prosthesis after self-expansion. Finally, postimplant balloon valvuloplasty was performed to ensure that the J-Valve fully fit within the failed prosthesis.

## References

[1] Nkomo VT Gardin JM Skelton TN Gottdiener JS Scott CG Enriquez-Sarano M. Burden of valvular heart diseases: a population-based study. Lancet. (2006) 368:1005–11. 10.1016/S0140-6736(06)69208-8 16980116

[2] Thourani VH Weintraub WS Guyton RA Jones EL Williams WH Elkabbani S et al. Outcomes and long-term survival for patients undergoing mitral valve repair versus replacement: effect of age and concomitant coronary artery bypass grafting. Circulation. (2003) 108:298–304. 10.1161/01.CIR.0000079169.15862.13 12835220

[3] Jones JM O'Kane H Gladstone DJ Sarsam MA Campalani G MacGowan SW et al. Repeat heart valve surgery: risk factors for operative mortality. J Thorac Cardiovasc Surg. (2001) 122:913–8. 10.1067/mtc.2001.116470 11689796

[4] Jamieson WR Burr LH Miyagishima RT Janusz MT Fradet GJ Lichtenstein SV et al. Reoperation for bioprosthetic mitral structural failure: risk assessment. Circulation. (2003) 108 Suppl 1:II98–102. 10.1161/01.cir.0000089184.46999.f4 12970216

[5] Guerrero M Vemulapalli S Xiang Q Wang DD Eleid M Cabalka AK et al. Thirty-day outcomes of transcatheter mitral valve replacement for degenerated mitral bioprostheses (valve-in-valve), failed surgical rings (valve-in-ring), and native valve with severe mitral annular calcification (valve-in-mitral annular calcification) in the united states: data from the society of thoracic surgeons/american college of cardiology/transcatheter valve therapy registry. Circ Cardiovasc Interv. (2020) 13:e008425. 10.1161/CIRCINTERVENTIONS.119.008425 32138529

[6] Takagi H Hari Y Kawai N Ando T. A meta-analysis of valve-in-valve and valve-in-ring transcatheter mitral valve implantation. J Interv Cardiol. (2018) 31:899–906. 10.1111/joic.12564 30311264

[7] Whisenant B Kapadia SR Eleid MF Kodali SK McCabe JM Krishnaswamy A et al. One-year outcomes of mitral valve-in-valve using the SAPIEN 3 transcatheter heart valve. JAMA Cardiol. (2020). 10.1001/jamacardio.2020.2974 32745164 PMC7391176

[8] Pirelli L Hong E Steffen R Vahl TP Kodali SK Bapat V. Mitral valve-in-valve and valve-in-ring: tips, tricks, and outcomes. Ann Cardiothorac Surg. (2021) 10:96–112. 10.21037/acs-2019-mv-169 33575180 PMC7867421

[9] Liu H Yang Y Wang W Zhu D Wei L Guo K et al. Transapical transcatheter aortic valve replacement for aortic regurgitation with a second-generation heart valve. J Thorac Cardiovasc Surg. (2018) 156:106–16. 10.1016/j.jtcvs.2017.12.150 29525255

[10] Zhu L Guo Y Wang W Liu H Yang Y Wei L et al. Transapical transcatheter aortic valve replacement with a novel transcatheter aortic valve replacement system in high-risk patients with severe aortic valve diseases. J Thorac Cardiovasc Surg. (2018) 155:588–97. 10.1016/j.jtcvs.2017.09.015 28992963

[11] Liu H Liu S Lu Y Wang W Yang Y Zhu L et al. Transapical transcatheter aortic valve implantation for predominant aortic regurgitation with a self-expandable valve. J Thorac Dis. (2020) 12:538–49. 10.21037/jtd.2020.01.04 32274119 PMC7139073

[12] Wei L Liu H Zhu L Yang Y Zheng J Guo K et al. A New Transcatheter aortic valve replacement system for predominant aortic regurgitation implantation of the J-Valve and early outcome. JACC Cardiovasc Interv. (2015) 8:1831–41. 10.1016/j.jcin.2015.08.021 26604056

[13] Nishimura RA Otto CM Bonow RO Carabello BA Erwin JP Guyton RA et al. 2014 AHA/ACC guideline for the management of patients with valvular heart disease: executive summary: a report of the American College of Cardiology/American Heart Association Task Force on practice guidelines. Circulation. (2014) 129:2440–92. 10.1161/CIR.0000000000000029 24589852

[14] Stone GW Adams DH Abraham WT Kappetein AP Généreux P Vranckx P et al. Clinical trial design principles and endpoint definitions for transcatheter mitral valve repair and replacement: part 2: endpoint definitions: a consensus document from the mitral valve academic research consortium. J Am Coll Cardiol. (2015) 66:308–21. 10.1016/j.jacc.2015.05.049 26184623

[15] Yoon SH Whisenant BK Bleiziffer S Delgado V Dhoble A Schofer N et al. Outcomes of transcatheter mitral valve replacement for degenerated bioprostheses, failed annuloplasty rings, and mitral annular calcification. Eur Heart J. (2019) 40:441–51. 10.1093/eurheartj/ehy590 30357365

[16] Bapat VV Khaliel F Ihleberg L. Delayed migration of Sapien valve following a transcatheter mitral valve-in-valve implantation. Catheter Cardiovasc Interv. (2014) 83:E150–4. 10.1002/ccd.25076 23784983

[17] Wang DD Eng M Greenbaum A Myers E Forbes M Pantelic M et al. Predicting LVOT obstruction after TMVR. JACC Cardiovasc Imaging. (2016) 9:1349–52. 10.1016/j.jcmg.2016.01.017 27209112 PMC5106323

[18] Wang DD Eng MH Greenbaum AB Myers E Forbes M. Karabon k. Validating a prediction modeling tool for left ventricular outflow tract (LVOT) obstruction after transcatheter mitral valve replacement (TMVR) Catheter. Cardiovasc Interv. (2018) 92:379–87. 10.1002/ccd.27447 29226591

[19] Little SH Bapat V Blanke P Guerrero M Rajagopal V Siegel R. Imaging guidance for transcatheter mitral valve intervention on prosthetic valves, rings, and annular calcification. JACC Cardiovasc Imaging. (2020) 14:22–40. 10.1016/j.jcmg.2019.10.027 32771581

[20] Asgar AW. Transcatheter mitral valve-in-valve-A plausible option but questions remain. JAMA Cardiol. (2020). 10.1001/jamacardio.2020.2993. [Epub ahead of print]. 32936231

